# The effect of Stage I-II periodontitis on the mechanical, structural, and biological properties of leukocyte and platelet-rich fibrin

**DOI:** 10.1590/1678-7765-2025-0483

**Published:** 2026-03-09

**Authors:** Sarvar Ibrahimli, Meltem Karsiyaka Hendek, Kubilay Baris, Ebru Olgun

**Affiliations:** 1 Kirikkale University Faculty of Dentistry Department of Periodontology Kirikkale Turkey Kirikkale University, Faculty of Dentistry, Department of Periodontology, Kirikkale, Turkey.

**Keywords:** Leukocyte, Periodontitis, Platelet-rich fibrin, Tensile strength

## Abstract

**Objective:**

This study aimed to compare the mechanical, biological, and structural properties of a leukocyte- and platelet-rich fibrin (L-PRF) membrane in periodontally healthy patients and those with periodontitis. A total of 30 individuals (17 men and 13 women) with a median age of 41 years were divided into two groups as periodontally healthy (control group) and as having periodontitis (test group).

**Methodology:**

Blood samples were collected and centrifuged at 2700 rpm for 12 minutes. A tensile test was performed to determine the mechanical properties of L-PRF membranes. Elasticity-modulus, tensile strength, and stretch at rupture were calculated. Platelet and leucocyte counts and ratios were calculated. Fibril thickness and density were evaluated by scanning electron microscopy imaging. The comparisons between groups were analyzed by the Mann–Whitney U test, and a p<0.05 value was considered statistically significant.

**Results:**

In the comparison of the control group versus the test group, pocket depth (3.55 (1.59-3.97) vs. 1.52 (1.47-3.53)), clinical attachment level (3.11 (1.77-4.23) vs. 0.06 (0.05-3.68)), plaque index (1.63 (0.05-1.75) vs. 0.04 (0.04-1.58)), gingival index (1.55 (0.04-1.63) vs. 0.05 (0.04-1.49)), and bleeding on probing (56.25 (3.7-70.11) vs. 3.57 (2.67-47.12)) were statistically significantly higher in the test group than in the control group (p= 0.009, p=0.008, p=0.008, p=0.009, p=0.009, respectively). Elasticity-modulus, tensile strength, and stretch at rupture values showed no statistically significantly differences between groups (p=0.745, p=0.754, p=0.602, respectively). Fibril thickness and density values showed no statistically significant differences between groups (p=1.000, p=0.753, respectively). Platelet count and platelet and leukocyte ratio values showed no statistically significant differences between groups (p=0.754, p=0.600, p=0.142, respectively). Leukocyte count was found to be statistically significantly higher in the control group than in the test group (p=0.028).

**Conclusion:**

This study showed no effect of Stage I-II periodontitis on the mechanical, structural, and biological properties of L-PRF. Further studies with larger sample sizes are required to confirm these findings.

## Graphical abstract

**Figure:**
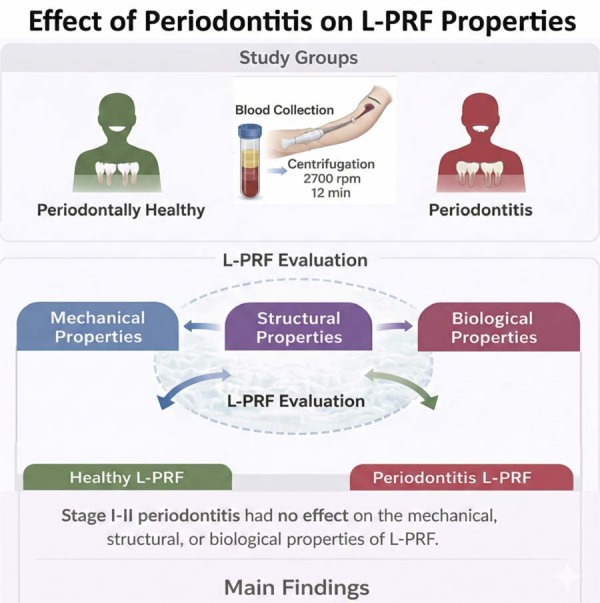

## Introduction

Periodontitis, a chronic inflammatory disease, progressively destroys the gingiva, cementum, periodontal ligament, and alveolar bone, primarily driven by hosts’ immune-inflammatory response to dental plaque biofilm. Microbial dental plaque and host and environmental factors (e.g., smoking and diabetes mellitus) are important factors in the development of periodontal disease.^1^ Evidence shows that periodontitis can affect the whole body of hosts, with periodontal pathogens entering the bloodstream and causing a systemic inflammatory response.^2^ Periodontal infection and inflammation have been shown to affect the development and severity of various systemic diseases and conditions.^3,4^ In addition to these diseases and conditions, periodontal disease has been shown to affect various cellular and molecular components of the blood, including leukocyte, erythrocyte, and platelet counts and acute-phase protein, immunoglobulin, and immunomediator levels.^5^The systemic inflammatory burden associated with periodontitis has been reported to influence specific pathways of bone marrow differentiation and to impair erythropoietic activity.^6^ The systemic inflammatory response induced by periodontitis may alter hematopoiesis, affecting blood cell counts and functions and potentially impacting the management and prognosis of hematological diseases.^7^ Elevated leukocyte, lymphocyte, red blood cell, mean corpuscular volume, platelet count, and neutrophil-to-lymphocyte ratio levels may serve as indicators of the heightened inflammatory response and tissue-destructive characteristics associated with periodontitis.^8^ In another study, a decrease in mean platelet volume was shown to be associated with severe periodontal inflammation, and a reverse shift in mean platelet volume values was observed following periodontal therapy, suggesting that mean platelet volume is linked to the inflammatory status of patients with severe periodontitis.^9^

Platelets play a vital role in homeostasis and wound healing, and thanks to their growth factors and cytokines stand among the cells with potential for regenerative therapy.^10^ Platelet concentrations, when used in surgical procedures involving hard and soft tissue, provide a controlled release of their intense cytokines and growth factors.^1 1^ Moreover, due to their autogenous origin, their primary advantage lies in the absence of immunological reactions and risk of infection. This has increased interest in the use of platelets and platelet-rich plasma in regenerative therapy. Various concentrations of platelets have been obtained from the past to the present day, and their positive contribution to the healing process has been observed when these products are used alone or in combination with other biomaterials.^12^Leukocyte- and platelet-rich fibrin (L-PRF) is an autogenous biomaterial that is rich in leukocytes and platelets derived by centrifugation of patients’ own blood, which contains no foreign substances or clotting factors in its preparation and content.^13^ The three main components of L-PRF—host cells, a three-dimensional matrix structure ,and growth factors—have been recorded as key elements in tissue regeneration. Research has shown that each of these three separate components of tissue regeneration is important during wound healing with L-PRF.^14^ L-PRF can also be considered as a biological three-dimensional network. It acts as a scaffold for cell migration and the proliferation, differentiation, and the delivery of growth factors. Platelets are massively retained in the fibrin network, and, by retaining growth factors in this three-dimensional fibrin network, it provides slow and gradual release over time.^15^ Since L-PRF was first described, a growing body of research has focused on examining its biological properties and evaluating methods for its improvement.^16,17^

Hutter, et al.^18^ (2001) investigated whether patients with periodontitis showed signs of anemia, showing that patients with periodontitis had lower hematocrit, erythrocyte, and hemoglobin counts and higher erythrocyte sedimentation rates. In Christan, et al.^19^ (2002), the effect of a non-surgical treatment on leukocyte counts and differential blood counts in smokers and non-smokers with generalized aggressive periodontitis was investigated. It was observed that leukocyte, neutrophil, and platelet counts decreased in non-smokers after periodontal treatment (but only platelet counts decreased in smokers).^19^

In the light of all this information, the aim of our study was to compare the mechanical, biological, and structural properties of L-PRF membranes in periodontally healthy individuals and those living with periodontitis. This study hypothesized that periodontitis influences the biological and structural properties of L-PRF membranes, with differences in the fibrin network architecture and cellular composition between individuals with and without periodontitis.

## Methodology

This cross-sectional study was conducted by including individuals who applied to the Kirikkale University Faculty of Dentistry, Department of Periodontology, for treatment and examination from January to August 2024. This study was approved by the Kirikkale University Clinical Research Ethics Committee under approval number [28.12.2023 Decision No: 27/01]. Written and verbal consent was obtained from each individual in this study.

### Groups

**Figure 1:**
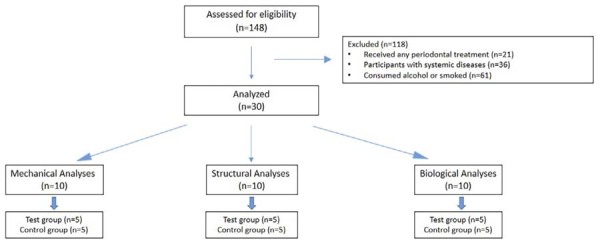
Flow diagram of patient selection and group distribution.

Individuals aged from 18 to 65 years who were systemically healthy and had received no periodontal treatment in the last six months were included in the study, whereas participants with systemic diseases associated with inflammation (such as diabetes mellitus or rheumatoid arthritis) and those on regular medication, who consumed alcohol or smoked, or were pregnant or breastfeeding were excluded from the study. After a clinical and radiographic examination, 30 individuals were divided into two groups as periodontally healthy (control group) and as having periodontitis (test group).^20^ Each group was divided into subgroups of five individuals for the mechanical, structural, and biological analyses (Figure 1).

### Periodontal clinical measurements

The periodontal status of all included individuals was determined by evaluating their pocket depth, clinical attachment level, plaque index,^21^ gingival index^22^ and bleeding on probing. Pocket depth and clinical attachment level were measured in six regions of all teeth. These values were averaged for each tooth. Regarding bleeding on probing, a periodontal probe was moved into the pocket. After approximately 20 seconds, the presence of bleeding was evaluated. In case of bleeding, the score of that region was recorded as (+), if not, it was recorded as (-). Measurements were made from four regions for each tooth. The percentage of bleeding was calculated by dividing the total number of bleeding areas by four times the number of teeth and multiplying by 100. A single examiner (S.I.), who had undergone calibration before this study, performed all clinical measurements.

### Collection of blood samples

Blood was collected from patients’ forearm vein into 9-ml empty plastic tubes containing glass particles (HEMA TUBE, Turkey). A blood sample was taken for mechanical and structural analyses, and two blood samples were taken for biological analyses. All blood samples were centrifuged within 60–90 seconds after collection to prevent premature clotting and ensure reproducibility. The blood samples were centrifuged at 400-g centrifugal force at 2700 rpm for 12 minutes (Hettich EBA 20 Centrifuge).^23^ L-PRF was obtained after centrifugation. L-PRF membranes were prepared using a PRF box by the same researcher under identical conditions. Pressure was applied consistently with gentle compression for 10 minutes to remove excess serum without damaging the fibrin matrix.

### Mechanical analyses

All mechanical tests were conducted using a universal testing machine (Utest, Ankara, Turkey). PRF membranes (1 mm thick, size 5 mm x 20 mm, rectangular) were fixed between two clamps with consistent gauge. A uniaxial tensile force was applied at a constant crosshead speed of 5 mm/min until failure. All samples were tested under the same ambient temperature and humidity conditions to avoid variability. Prior to each test, the device was calibrated, and all specimens were aligned manually to avoid pre-loading or slippage. Such standardization ensured consistency across samples. The obtained data were evaluated by a blinded researcher on a stress-strain curve. Elasticity- (E) modulus, tensile strength, and stretch at rupture were calculated. Prior to data analysis, all samples were assigned anonymous numerical identifiers by an independent investigator who had no involvement in the evaluation procedures. The blinded researcher only had access to these identifiers, remaining unaware of the group allocation of each sample. To prevent order-related bias, the sequence in which the samples were tested and analyzed was randomized using a coin-toss method.

### Structural analyses

**Figure 2:**
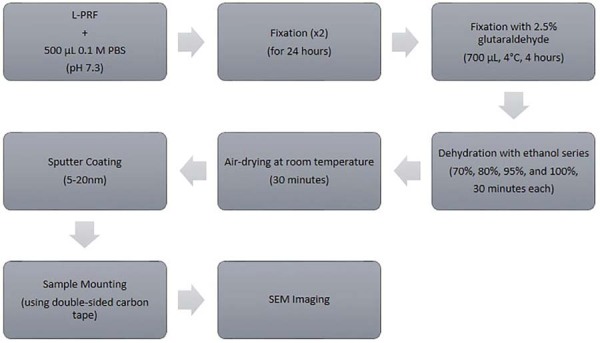
Fibrin measurement workflow (residue and exudate subtraction).

Immediately after preparation, the L-PRF membranes were transferred to 500 μL of a 0.1-M phosphate-buffered saline (PBS, pH 7.3) and placed on stubs to prevent adhesion. The samples were fixed for 24 hours. This procedure was repeated twice. Subsequently, the membranes were immersed in 700 μL of 2.5% glutaraldehyde at 4°C for 4 hours, followed by dehydration by a graded ethanol series (70, 80, 95, and 100% — 30 minutes each). After dehydration, the samples were air-dried at room temperature for 30 minutes to remove the residual moisture. To enhance imaging resolution and electrical conductivity, the surfaces were sputter-coated with a thin layer of gold/palladium (5–20 nm) using a Polaron sputter coater, avoiding excessively thick coating. The membranes were mounted on stubs with double-sided carbon tape and thoroughly dried to ensure stability under vacuum. Scanning electron microscopy was performed using a JEOL JSM 5600 instrument with a secondary electron detector at an acceleration voltage of 20 kV to minimize sample heating and evaporation (Figure 2). Surface morphology was examined at 10,000× magnification by a blinded researcher who was unaware of the sample groups. For each membrane, images were acquired from consistent, randomized regions across the surface to ensure representative measurements of fibril morphology and density. Fibril thickness and density were quantified on ImageJ (NIH, version 5.3), with three micrographs analyzed per sample. Fibrin thickness was determined by measuring at least 40–50 fibers per image, the mean values of which were calculated for each sample.

### Biological analyses

To calculate cell counts, two tubes of blood samples were taken from each patient, one containing heparin lithium and another with a vacuum tube without an anticoagulant. The blood in the heparin lithium-containing tube was reserved for complete blood count. The blood sample volume in the non-anticoagulated tube was marked with an acetate pen and centrifuged. After centrifugation, the L-PRF clots were removed from the tubes. The residue in the tubes was diluted with saline to the marked volume and shaken gently. After compressing the L-PRF clots, the L-PRF exudate was collected with a syringe and transferred to another anticoagulant-free tube, which was diluted with saline to the marked volume. The tubes were shaken to obtain a homogenous mixture. Blood counts were taken from the tubes containing residue and exudate and from the heparinized tube and transferred to Eppendorf tubes. To prevent crystal formation inside the cells, 10% dimethyl sulfoxide was added to each Eppendorf tube. Cell residue, exudate, and whole blood sample counting was performed with a hematology analyzer (Mindray BC-6800 Hematology Analyzer). The cell content of the L-PRF membranes was evaluated by subtracting the sum of the cells in the residue and exudate from the whole blood cells in the heparinized tube. Platelet and leucocyte counts were measured by a blinded investigator. Platelet and leucocyte ratio was calculated by dividing the number of platelets and leukocytes in L-PRF by the number of leucocytes and platelets in whole blood and multiplying by 100.

### Statistical analysis

Based on preliminary data and an effect size of 6.9, we calculated that a sample of three participants per subgroup would provide 95% power to detect significant differences. Fiber diameter was used as the primary parameter to estimate the effect size under the same experimental conditions as those in this study. The obtained data were statistically analyzed on SPSS for Windows, ver. 26 (SPSS Inc., Chicago, ILL, USA). Descriptive statistics of the numerical data were reported as medians (interquartile range) (IQR) or means ± standard deviation, depending on the normal distribution of the data. Frequency and percentage values were calculated for gender. The examination of the measurements in each group was analyzed by the Mann-Whitney U test. Gender distribution between groups was analyzed by the chi-squared test. A p<0.05 value was considered statistically significant.

## Results

The ages of the individuals in this study ranged from 23-55 years. A total of 30 individuals (17 men and 13 women) were included. Age and gender distributions were similar between the groups. Patients with periodontitis were diagnosed as having generalized stage I-II Grade B periodontitis. The medians of all evaluated periodontal clinical parameters were statistically significantly higher in the test group than in the control group (p<0.05) (Table 1).

**Table 1:** Demographic characteristics and clinical parameters of groups.

	Control Group	Test Group	P value
	**(n=15)**	**(n=15)**	
Age (years; (median (IQR))	38 (31-44)	43 (36-50)	0.169
Sex (n)			
Female/Male	05/10	08/07	0.262
PD (mm) (median (IQR))	1.52 (1.47-3.53)	3.55 (1.59-3.97)	0.009
CAL (mm) (median (IQR))	0.06 (0.05-3.68)	3.11 (1.77-4.23)	0.008
PI (median (IQR))	0.04 (0.04-1.58)	1.63 (0.05-1.75)	0.008
GI (median (IQR))	0.05 (0.04-1.49)	1.55 (0.04-1.63)	0.009
BOP (%) (median (IQR))	3.57 (2.67-47.12)	56.25 (3.7-70.11)	0.009
Stage I Grade B Periodontitis (n)	-	6	
Stage II Grade B Periodontitis (n)	-	9	

PD: Pocket depth; CAL: Clinical attachment level; PI: Plaque index; GI: Gingival index BOP: Bleeding on probing

Chi-square test for gender distribution

Mann Whitney U test for intergroup comparison

### Mechanical tests

For mechanical analyses, five samples per group were utilized. The E-modulus was 0.06 (0.06-0.08) MPa in the control group and 0.05 (0.05-0.09) MPa in the test group. The tensile strength was 0.10 (0.09-0.15) MPa in the control group and 0.14 (0.07-0.19) MPa in the test group. The stretch at rupture was 1.72 (1.57-1.99) mm in the control group and 2.02 (1.21-2.41) mm in the test group. E-modulus, tensile strength, and stretch at rupture values were statistically insignificant between groups (p=0.745, p=0.754, p=0.602, respectively) (Table 2).

**Table 2:** Comparison of mechanical, structural, and biological parameters in groups.

	Control Group	Test Group	P value
**Mechanical parameters**	**(n=5)**	**(n=5)**	
E-modulus (MPa) (median (IQR))	0.06 (0.06-0.08)	0.05 (0.05-0.09)	0.745
Tensile strength (MPa) (median (IQR))	0.10 (0.09-0.15)	0.14 (0.07-0.19)	0.754
Stretch at rupture (mm) (median (IQR))	1.72 (1.57-1.99)	2.02 (1.21-2.41)	0.602
**Structural parameters**	**(n=5)**	**(n=5)**	
Fibril thickness (µm) (median (IQR))	0.42 (0.21-0.51)	0.33 (0.21-0.54)	1.000
Fibril density (%) (median (IQR))	99.72 (92.46-100)	99.65 (97.25-99.89)	0.753
**Biological parameters**	**(n=5)**	**(n=5)**	
Platelet count (10^3^/µL) (median (IQR))	103 (47-148)	108 (78-132)	0.754
Platelet ratio (%) (median (IQR))	80 (71-90)	86 (81-92)	0.600
Leukocyte count (10^3^/µL) (median (IQR))	3.86 (3.35-4.06)	2.54 (2.22-3.29)	0.028 *
Leukocyte ratio (%) (median (IQR))	53 (50-60)	42 (38.5-57)	0.142

Mann Whitney U test for intergroup comparison

*Significant difference between groups

### Structural tests

**Figure 3:**
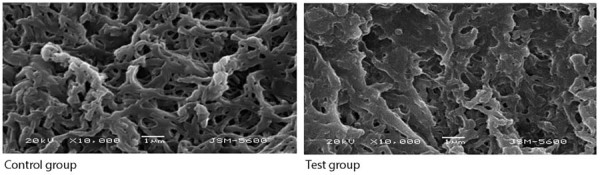
Scanning electron microscopy images of control and test groups at 10000× magnification.

In total, five samples from each group were used for structural analyses. The fibril thickness totaled 0.42 µm (0.21-0.51) in the control group and 0.33 µm (0.21-0.54) in the test group. Fibril density equaled 99.72% (92.46-100) in the control group and 99.65% (97.25-99.89) in the test group. No statistically significant difference was found between the groups regarding fibril thickness and density (p = 1.000, p = 0.753, respectively) (Table 2) (Figure 3).

### Biological tests

In total, five samples per group were used for biological analyses. The platelet ratio totaled 80% (71-90) in the control group and 86% (81-92) in the test group. Platelet count equaled $103\times 10^{3}/\mu L(47-148)$ in the control group and $108\times 10^{3}/\mu L(78-132)$ in the test group. Platelet ratios and counts were statistically insignificant between groups (p = 0.600, p = 0.754, respectively). Leukocyte ratios equaled 53% (50-60) in the control group and 42% (38.5-57) in the test group. The groups showed no significant differences regarding leukocyte ratios (p = 0.142). Leukocyte counts totaled $3.86\times 10^{3}/\mu L(3.35-4.06)$ in the control group and $2.54\times 10^{3}/\mu (2.22-3.29)$ in the test group. A significant difference was found between the groups regarding leukocyte count (p = 0.028). Leukocyte counts in the control group were significantly higher than that in the test group (Table 2).

## Discussion

This study aimed to evaluate the mechanical, biological, and structural properties of L-PRF membranes in individuals with and without periodontitis. We hypothesized that periodontal health status affects the biological and structural features of L-PRF membranes, including variations in fibrin network organization and cellular content. We observed that the presence of Stage I-II periodontitis fails to compromise the mechanical, structural, and biological properties of L-PRF.

The platelet-rich fibrin clot and membrane have a complex and unique structure with a natural fibrin matrix, platelet aggregates, leukocytes, growth factors, and cytokines. Each of these components plays a different role in inflammation and wound healing. The biological properties of PRF depend on its three-dimensional structure and the levels of growth factors and cytokines in platelets. Fibrin architecture and leukocyte content affect cytokine release.^24,25^ Various platelet concentrations, developed from past to present, have been applied in numerous fields of dentistry, and their effects are attributed to factors such as growth factors, cell counts, and the quantity and density of the fibrin network.^11^ In general, an intact gingival epithelium acts as a natural physical barrier against the systemic spread of oral bacteria. However, in the presence of periodontal inflammation, periodontal bacteria and their products can enter periodontal tissues and circulation, mostly through ulcerated and disrupted sulcular epithelium. Several inflammatory markers in inflamed periodontal tissues can enter the bloodstream and contribute to the inflammatory burden. This may explain the relationship between periodontal disease and systemic status.^26^ Therefore, the mechanical, structural, and biological properties of L-PRFs obtained from patients with periodontitis and periodontally healthy individuals were compared in this study.

Several factors can affect the fibrin network of L-PRF membranes.^27,28^ After blood collection, platelets are activated as soon as they touch the wall of the blood collection tube, which triggers the coagulation cascade and stimulates the conversion of fibrinogen to fibrin by circulating thrombin. Initially, fibrinogen is concentrated in the upper part of the tube. However, fibrinogen collects in the middle part of the tube during centrifugation. Therefore, if the time interval between blood collection and the start of centrifugation is too long, fibrinogen fails to condense and fibrin polymerizes in a diffuse manner.^14^ This time interval should be kept as short as possible (ideally from 60 to 90 seconds).^29^ Decreasing the centrifugation speed and time also leads to a decrease in fibrin network density. Therefore, the values of these factors were kept similar between the groups in our study.

In a study comparing the mechanical and chemical properties of membranes with different platelet concentrates (L-, advanced-PRF, and titanium-prepared-PRF), it was observed that T-PRF had the greatest tensile strength and modulus of elasticity, whereas advanced platelet-rich fibrin (A-PRF) consistently released growth factors for longer; a significant difference was found between the groups.^30^ A similar study concluded that A-PRF showed a significantly greater maximum tensile strength score and a higher mean tensile strength than L-PRF.^16^ In another study comparing the mechanical properties of several PRF membranes based on tensile strength, it was concluded that A-PRF+ showed superior viscoelastic resistance under opposing tensile forces.^17^ Therefore, in this study, it was thought that using a single type of PRF instead of varying platelet concentrations prepared with different protocols would be appropriate for its purpose. L-PRF, which is frequently used in studies, was preferred.

In a study investigating the effect of diabetes status on the mechanical and biological properties of PRF, it was reported that A-PRF membrane from non-diabetic individuals had better tensile strength, tension, and growth factor release, whereas well-controlled diabetic individuals had higher growth factor releases.^31^ In another study examining the effect of cigarette smoking on the mechanical and biological properties of L- and A-PRF membranes, smoking was shown to affect platelet activation and affect the tensile strength of L-PRF membranes and growth factor release from A-PRF membranes in smokers, although statistically insignificant.^32^

Moreover, studies evaluating the effect of demographic data such as age and gender on PRF reported that women showed more fibrinogen and growth factor release, whereas men showed greater Young’s moduli in compression tests.^33^ However, it has been reported that platelet and leukocyte counts decrease in L-PRF membranes with increasing age.^34^ The similar age and gender distribution of our groups helped to eliminate the differences arising from these demographic data.

In a methodologically similar investigation assessing the influence of diabetes and periodontitis on the tridimensional structural properties of PRF membranes and the spatial distribution of growth factors within the plasma and erythrocyte ends of the PRF matrix, researchers found that PRF obtained from subjects with coexisting conditions was markedly more porous than that from healthy participants or those with a single inflammatory disease.^35^ The erythrocyte end of the PRF membrane has been observed to have finer fibers that are arranged in a relatively denser and compact structure with smaller porosities when compared to the plasma end. Periodontitis is a chronic bacterial infection of the oral cavity that elicits local inflammation. The resultant cytokines and inflammatory mediators can enter the bloodstream, amplifying systemic inflammation. In our study, the similarity in PRF density and thickness between periodontitis patients and healthy individuals may indicate that systemic response seems to be related to the severity of the disease. In other words, periodontitis (stage I–II) likely exerts a milder effect than that of the systemic impact of diabetes.

In a study examining the potential effect of antithrombotics on L-PRF membranes, it was shown that L-PRF membranes from patients using drugs were weaker, less elongated and with fewer leukocytes than the L-PRF membranes of patients taking none such drugs.^36^ In an in vitro study investigating the mechanical properties of L-PRF membranes, fibrin structure, cellular content, and the effect of anticoagulant treatment on L-PRF, it was reported that low doses of anticoagulant failed to affect the mechanical properties, fibrin network, or the cellular content of L-PRF, whereas high doses disrupted L-PRF formation.^37^ The fact that the mechanical structural and biological properties of PRF showed no differences between the groups in our study may be due to the inclusion of individuals with mild/moderate periodontitis in parallel with the drug dose in this study.

Leukocytes, or white blood cells, are vital to our immune system, protecting the body from infections and foreign invaders such as bacteria and viruses. Leukocyte count is measured to determine whether an infectious process is present—it should range from 5000 to 10,000 cells/mm^3^. Although leukocyte levels differed between groups regardless of the presence of periodontal disease in participants, this difference was clinically insignificant. In both groups, leukocyte levels remained below pathological thresholds. This condition may be associated with the inflammatory burden in individuals with periodontal disease.

Although platelet counts and concentrations are critical determinants of PRF quality, these parameters largely depend on individuals’ systemic hematological status rather than on local periodontal inflammation. Leukocyte recruitment and local inflammatory mediator activity are primarily affected by periodontitis, whereas systemic platelet production and circulating platelet counts remain largely unchanged. Potential technical variability was minimized by applying standardized blood collection and centrifugation protocols to all groups. Therefore, the lack of significant differences in platelet count and ratio between the groups is consistent with these considerations.

This study has certain limitations, such as its lack of information concerning the timing of disease onset and its omission of subjects showing advanced periodontal pathology. Moreover, the generalizability of the findings of the study is limited due to the small sample size; further research with larger samples is needed.

## Conclusion

In conclusion, considering its limitations, this study showed no significant differences in the mechanical, structural, or biological characteristics of L-PRF between periodontally healthy and stage I–II periodontitis groups. Future studies should increase their sample sizes and use large cohort studies under varying preparation protocols and PRF variants that include different stages of periodontitis and consider the extent and severity of the disease.
